# Balancing care demands and personal needs: A typology on the reconciliation of informal dementia care with personal life based on narrative interviews

**DOI:** 10.1177/14713012211008306

**Published:** 2021-04-13

**Authors:** Sophie Gottschalk, Lydia Neubert, Hans-Helmut König, Christian Brettschneider

**Affiliations:** Department of Health Economics and Health Services Research, 37734 University Medical Center Hamburg-Eppendorf, Hamburg Center for Health Economics, Germany

**Keywords:** dementia, qualitative, typology, reconciliation, balancing needs, informal care

## Abstract

Finding a balance between care and personal needs is often challenging for individuals caring for a person with dementia. The present study aimed to understand how primary and secondary family carers of people with dementia perceive the reconciliation of informal care and their personal life throughout the course of care. Narrative interviews with 14 carers (*n*=10 female, mean age: 48.79) from seven care networks were conducted. The interviews were analysed using the documentary method. The result was a relational type formation which comprises different process types: ‘Respect your boundaries, stay calm, but plan ahead’, ‘Acting strategic is making it work’, ‘Reflect the situation, deal with it, and pull it through’ and ‘There is no alternative, you have to live with it’. This typology was based on two related dimensions: *the way people negotiate their own needs against the demands of care* (prospective, strategic, reflective and limited) and the resulting *attitudes towards reconciling informal care and personal life* (accepting, withstanding and enduring). The results yield implications for practice, such as considering individual needs and the care network to provide effective support and promoting the importance of self-care behaviours to achieve a balance between care and personal life.

## Introduction

Life expectancy is increasing worldwide, so is the number of older people. Older age is associated with physical and cognitive decline which can affect the ability to perform everyday activities and thus often leads to a need for support. In Germany, 2.59 million people are cared for at home, of whom 1.76 million receive care by relatives only (informal care) (Federal Statistical Office (Destatis) (2018). A group of older adults who have a particularly high need for care are people with dementia. To date, Germany counts 1.63 million people with dementia (Deutsche Alzheimer Gesellschaft e.V., 2018). As the population ages, this number will increase (Alzheimer’s Disease International, 2018), and consequently, the demand for informal care will also increase.

The effects of informal care on the carer have been subject of many studies. There is a strong consensus that providing informal care, especially for people with dementia, can be burdensome and therefore can negatively affect mental and physical health, as well as quality of life (Bauer & Sousa-Poza, 2015; Karg et al., 2018; Schulz et al., 2012; van der Lee et al., 2014). However, there is an emerging body of literature that opposes this overly negative picture of informal care (Carbonneau et al., 2010; Lloyd et al., 2016; Quinn & Toms, 2018; Roth et al., 2015). This literature shows that negative and positive effects of informal care (such as meaning of life, personal growth, development of new skills or maturity) are not mutually exclusive, but co-occur (Campos et al., 2019; Garcia-Mochon et al., 2019; Joling et al., 2016; Pendergrass et al., 2019; Pristavec, 2019; Quinn et al., 2019).

Although many different factors, for example, relationship quality between the carer and the person with dementia (Daley et al., 2017; Lloyd et al., 2016) and within the caring network (Neubert et al., 2020b), certainly play a role in whether or to what extent informal care is perceived as challenging, a common aspect appears to be that informal care interferes with other areas of personal life (e.g. social, occupational or family life). Previous studies indicated that the perception of care as burdensome is related to the perceived need to constantly oversee the person with dementia and to continuously coordinate his/her life, as well as to the lack of or dissatisfaction with social support (Benson et al., 2019; Brügger et al., 2016; Garcia-Mochon et al., 2019; Lopez Hartmann et al., 2019). This degree of commitment is time-consuming and results in restrictions in personal life, such as limited time to pursue one’s own interests, loss of freedom and feelings of loneliness or social isolation (Brügger et al., 2016; Large & Slinger, 2015; Ransmayr et al., 2018; Varik et al., 2019). Thus, finding a balance between meeting care demands and pursuing personal goals appears to be challenging for carers (Lopez Hartmann et al., 2016; Quinn et al., 2015; Rayment et al., 2019). A carer’s ability to deal with these issues depends on individual resources like coping abilities, personality traits and other psychological attributes (e.g. mastery, sense of competence), or the context of care (Chiao et al., 2015; Hansen & Slagsvold, 2013; Joling et al., 2016; Rayment et al., 2019; Roth et al., 2015). Since dementia is associated with psycho-behavioural symptoms and the demand for care typically increases as the disease progresses, carers constantly need to adapt their strategies and resources. Hence, challenges of caring for a person with dementia change over time. In a systematic review on the main factors of carer burden in informal dementia carers (Chiao et al., 2015), the need for studies that identify patterns throughout the course of caring is emphasised. Moreover, people with dementia are often cared for by more than one primary carer, yet little is known about the consequences of informal care on secondary carers (Barbosa et al., 2011; Gaugler et al., 2003). Recent findings, for example, indicate that secondary carers, despite providing a lower amount of care, also face clinically significant distress (Gonçalves-Pereira et al., 2020).

Therefore, the aim of the present study is to obtain an in-depth understanding of how primary and secondary informal family carers of a person with dementia perceive the reconciliation of informal care and their personal life and how these perceptions have changed throughout the course of caring. By doing so, we identify different patterns of challenges in caring for a person with dementia. To this end, we employed a constructivist approach by using the documentary method which was developed by Ralf Bohnsack and extended by Arnd-Michael Nohl (2010). With the term ‘personal life’, we refer to a person’s personal daily routine, personal interests, ideas and visions about one’s life, as well as to internal/emotional aspects linked to these aspects. In the current study, we deliberately did not focus on the areas of family and employment since these were reported elsewhere (Neubert et al., 2020a, 2020b).

## Methods

### Participants

This study was part of a mixed-methods pilot study which aimed to explore the reconciliation between caring for a person with dementia, family and employment from a network perspective (Neubert et al., 2018). In the original study, participants were deemed eligible if they were 18 years or older, German-speaking, had no cognitive impairments, verbally confirmed the dementia diagnosis of the cared-for person and were part of a care network with at least one (ideally two) other network members being available to participate in the study. Moreover, at least one person in the care network had to be employed. Owing to the network perspective that the original study was intended to take, not only primary carers, but also secondary carers were included.

Fourteen family carers (7 primary and 7 secondary carers) from 7 care networks (A–F) with a mean age of 48.79 years (ranging from 19 to 65 years) participated in the current study. The majority was female (*n* = 10) and provided care to a parent or parent-in-law (*n* = 10); the others cared for a grandparent (*n* = 3) or a spouse (*n* = 1) (Table 1). Among the 14 carers, 10 lived in an urban area and six were cohabiting with the person with dementia. The average health-related quality of life, measured by the EQ-5D-5L index, was 0.93 (±0.08), indicating very high health-related quality of life. In general, the perceived care-related quality of life, measured by the CarerQoL-7D index, was also high with a mean of 79.74 (±22.95) on a range between zero and 100; only one participant scored very low (10.6).

**Table 1.** Sociodemographic and psychographic characteristics of *N*=14 primary and secondary family carers of persons with dementia living in the community.

*n* = 14		
Age	Mean (range)	48.79 (19–65)
Female	*n* (%)	10 (71.4)
Relationship to the PwD	*n* (%)	
Spouse		1 (7.1)
Child		9 (64.3)
Grandchild		3 (21.4)
Daughter-in-law		1 (7.1)
Educational level	*n* (%)	
Vocational training degree		10 (71.4)
College degree		3 (21.4)
Other		1 (7.1)
Employed	*n* (%)	12 (85.7)
Living in the same household as the PwD	*n* (%)	6 (42.9)
Urban residential area	*n* (%)	10 (71.4)
EQ-5D-5 L index	Mean (*SD*.)	0.93 (0.08)
EQ-VAS	Mean (*SD*.)	77.5 (15.0)
CarerQoL-7D index	Mean (*SD*.)	79.7 (23.0)
ASKU (self-efficacy)	Mean (*SD*.)	4.33 (0.60)
PCI	Mean (*SD*.)	
Proactive coping (17–68)		47.14 (5.3)
Reflective coping (11–44)		34.07 (4.4)
Strategic planning (4–16)		12.14 (1.6)
Preventive coping (10–40)		27.43 (4.0)
Instrumental support seeking (8–32)		24.71 (4.7)
Emotional support seeking (5–20)		14.14 (2.6)
Avoidance coping (3–12)		6 (1.5)

PwD = person with dementia; EQ-5D-5L index = health-related quality of life, 0 indicating death, 1 indicating perfect health, negative values worth than death; EQ-VAS = health-related quality of life, rated on a visual analogue scale (0–100); CarerQoL-7D = Care-related Quality of Life instrument seven dimensions (0–100, higher scores indicating higher care-related quality of life); ASKU = general self-efficacy, short-form (1–5); PCI = Proactive Coping Inventory, higher scores indicating more frequent use of the respective coping strategy.

### Procedure

Ethical approval for this study was obtained from the German Society of Nursing Sciences (approval no. 17-006). All participants gave written informed consent to participate in the study. The participants were recruited using a purposive sampling strategy. Contact to potential participants was established via gatekeepers at counselling centres for older adults and informal carers in and around Hamburg (Germany). One care network from Southern Germany entered the study via a contact at a conference. The participants were interviewed individually by the second author between September 2017 and September 2018. Owing to the constructivist approach, a narrative interview (Schütze, 1976) was chosen which aims at eliciting stories of experiences. The participants were given a narrative stimulus (‘As you know, I am interested in how the care [of your ...] works in your family. But in order to be able to put the whole thing into perspective and to understand how it started, I would like to ask you to tell me the story of your family up to the present day’.). Depending on what was already addressed by the respondents themselves, the interviewer asked narrative-generating questions from a previously developed interview guide. The interviews took place at the carers’ homes or in a separate room at the University Medical Center Hamburg-Eppendorf and lasted between 30 and 150 min. The participants from Southern Germany were interviewed via Skype. Apart from the initial contact to ensure eligibility and to find an appointment for the interview, no relationship between the second author and the interviewees was established prior to the study. At the end of the interview, the participants were given a questionnaire to collect sociodemographic, psychographic and health-related data. The interviews were transcribed verbatim by the first author and corrected by a second listener (second author). The corrected transcripts were used in the data analysis. By transcribing, for example, emphases and pauses, the transcripts have a high information content which could be included in the interpretation. A detailed description of the development of the interview guide, the experience and training of the interviewer (second author), and the instrument selection for the questionnaire can be found in the study protocol (Neubert et al., 2018).

## Data analysis

The transcripts were analysed using the documentary method (Nohl, 2010) without using any specific software for the interpretation. This method aims to reconstruct the experiences and orientations of interviewees, their so-called *orientation frameworks*, and by doing so, to abstract their habitual action or practice. This is possible by identifying the *documentary meaning* of narratives which refers to *how* something was said and goes beyond summarising *what* has been said. Thus, this approach is suitable to answer our research questions. The documentary method comprises three different stages of interpretation: formulating interpretation (*what* was said), reflecting interpretation (*how* something was said) and type formation. The first and second author performed the first two stages independently; the type formation was undertaken jointly. Nohl describes different possibilities of type formation (Nohl, 2017). Since we were able to identify two mutually dependent dimensions (*orientation frameworks*) during the analysis, the relational type formation was applied in this study (Nohl, 2013). Throughout the process, the findings were regularly discussed between the first and second author and in an external interpretation group. The interpretation group comprised three interdisciplinary researchers working with the documentary method who regularly discussed their material; for example, the interpretation of excerpts from the transcripts or the validity of the type formation. In preparing this article, the Consolidated Criteria for Reporting Qualitative Research (COREQ) (Tong et al., 2007) and the Standards for Reporting Qualitative Research (SRQR) guidelines (O’Brien et al., 2014) have been followed. While COREQ was initially developed for focus groups and interviews and has an orientation towards grounded theory, SRQR covers a broader range of qualitative studies for which some COREQ criteria may be less relevant (Peditto, 2018).

## Findings

### Relational type formation

**Figure 1.**
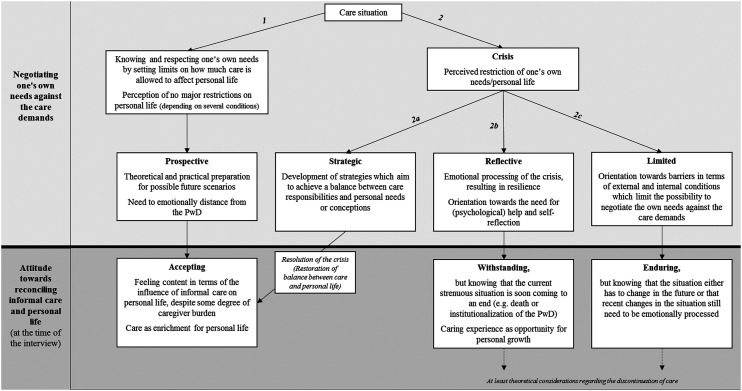
Process types (relational types) of family carers’ perceptions of reconciling informal care and personal life; results based on a qualitative study with *N* = 14 primary and secondary family carer of persons with dementia (PwD) living in the community.

All participants in this sample have provided care for their family member with dementia for several months or years, without being able to identify when exactly they first undertook the carer role. This indicates that caring is a dynamic state. We reconstructed a typology of four different process types (‘Respect your boundaries, stay calm, but plan ahead’, ‘Acting strategic is making it work’, ‘Reflect the situation, deal with it, and pull it through’ and ‘There is no alternative, you have to live with it’) that result from the relational type formation. This typology emerged from two mutually dependent dimensions. The first dimension in which the carers differed was in their way of *negotiating their own needs against the care demands*. From this dimension, we were able to reconstruct four different forms of negotiation processes: prospective, strategic, reflective and limited. Taking on a carer role initiated internal negotiation processes in all participants of this study. These negotiation processes refer to a conscious and unconscious balancing of different areas of personal life, roles and their respective demands (e.g. informal care, job, family and leisure time). Perceived balance between different areas of life is very subjective and depends on individual preferences and approaches to life. Hence, a perceived balance can be achieved even if caring for a person with dementia occupies a relatively large share of time compared to other areas of life. Unlike the first dimension which reflects a process over a non-specified period of time, the second dimension, the *attitudes towards reconciling informal care and personal life* as a result of the negotiations, constitutes a snapshot of the participants’ attitude at the time of the interview. These attitudes manifest themselves in accepting, withstanding or enduring the situation. The relation between these two dimensions also means that specific negotiation processes lead to specific attitudes towards reconciling informal care and personal life and vice versa; these attitudes may influence how future situations are negotiated. Graphical representations of the type formation can be found in Table 2 and Figure 1.

**Table 2.** Relational type formation of family carers’ perceptions of reconciling informal care and personal life; results based on a qualitative study with *N* = 14 primary and secondary family carers of persons with dementia living in the community.

Dimension 1: ‘Negotiating one’s own needs against the care demands’	Dimension 2: ‘Attitude towards reconciling informal care and personal life’		
	Accepting	Withstanding	Enduring
Prospective	‘Respect your boundaries, stay calm, but plan ahead’ (process type 1)		
Strategic	‘Acting strategic is making it work’ (process type 2a)		
Reflective		‘Reflect the situation, deal with it, and pull it through’ (process type 2b)	
Limited			‘There is no alternative, you have to live with it’ (process type 2c)

### Process type 1: ‘Respect your boundaries, stay calm, but plan ahead’

The first process type that emerged from the data comprised five, mainly secondary carers (*n* = 4). Three carers were adult children, and two were grandchildren (aged 21 and 24 years) of the person with dementia. Except from one grandchild, none of them was cohabiting with the person with dementia. Family carers of this type do not perceive major restrictions in their personal life which they achieve by knowing and respecting their own needs. They set clear limits on how far they are willing to allow the provision of care to affect their personal lives. They do what they are willing and able to do within their personal limits and explicitly name tasks they refuse to undertake.

*What I do (I don’t feel), is much a burden, in terms of time or anything else (as I said), with Online-Banking and everything, nowadays it’s not a problem at all. […] It’s only a bit of time. […] But I would really draw the line if she needs help with taking care of herself, washing herself, when such things start, if she were to refuse the help of formal carers, then I think I would say, so, now, I’m out.* ^*1*^ *[Although being the secondary carer, A2 feels responsible for tasks like washing the person with dementia. She does not take it for granted that her sister (A3, primary carer), takes over these tasks alone. However, despite in principle feeling responsible, she comes up against her limits when it comes to her mother’s personal hygiene.]*

*(A2, daughter, secondary carer, non-cohabiting)*

*So I’m actually more like the support behind everything, my mother takes care of grandpa, because I couldn’t do it. […] I think that she is also the more patient of the two of us, so in terms of that it wouldn’t make much sense, so then I can rather take on the tasks behind it.*

*(C3, granddaughter, secondary carer, cohabiting)*

Being able to preserve boundaries and to keep the influence of care on their personal lives at a low level depends on several conditions. Some of the carers have an advantaged care network which is characterised by, for example, a harmonious relationship between the network members who share care tasks or by having special expertise regarding the administration of care (Neubert et al., 2020a, 2020b). Moreover, the cared-for person’s preferences, character traits and extent of dementia support the reconciliation of informal care and personal life.

*All three of us, well I found that conversations or visits for more than half an hour are no longer possible. […] you notice that she [person with dementia] then also switches off; and then also becomes very quiet and only looks out the window (.) or says she is not well, that is a standard saying of hers, then I realize ok it’s enough and then I happily say goodbye.*

*(A1, son, secondary carer, non-cohabiting)*

Further conditions that facilitate the preservation of boundaries are part-time employment and the proximity or distance between the homes of the person with dementia and the carer. Carers in part-time employment find it easier to integrate informal care into their daily routines, in addition to their work, without feeling too much of a burden. Living near the person with dementia keeps the temporal expenditure of regular visits low, whereas living further away precludes regular visits, and hence, the regular daily schedule remains largely unaffected by informal care.

*Yes, so actually I try to go by every day; it is of course quite good due to the spatial proximity, […] really only across the street, therefore you can really do that. […] So if I had to get into the car and then drive to my mother for an hour and then be away from home for three hours, I wouldn’t do that.*

*(A3, daughter, primary carer, non-cohabiting)*

Moreover, the individuals of process type 1 are oriented towards respecting the wishes and ideas of the person with dementia and thereby taking the care, as well as the dementia-related behaviours, (as) calmly (as possible).

*If you say now I want to have a perfect household and take care of her perfectly, then I think it wouldn’t matter if you’re with others or alone in it, I think you’d run yourself into the ground. If you just see it in a bit more a relaxed way and try to see what her wants are, if she wants to live in such a way or dress in so and so a way, then that’s how it is.*

*(A2, daughter, secondary carer, non-cohabiting)*

The way they negotiate their needs and those of the person with dementia is *prospective*. They theoretically and practically prepare themselves for possible future scenarios (e.g. relocating the person with dementia to a long-term care home).

*We know that there is no other way. […] That means that one of us [because of a sister’s Sabbatical] completely drops off, and us others can’t really fill in the gaps. It could be that in that time [we put her into daycare] (.) she completely rejects daycare but inpatient care can happen. We have to see how she holds up until that point and how the dementia progresses. We’ll see. But that is like the next big hurdle that we know of, that we can’t avoid? But it’s coming.*

*(A1, son, secondary carer, non-cohabiting)*

For some carers, this is accompanied by a need to emotionally distance from the person with dementia in order to reduce their own emotional burden and facilitate rational decision making which further facilitates accepting the situation and taking it calmly.

*For me, my mother has died long ago. It’s not my mother I’m visiting, it’s her shell. So I have to say, so, um, (.) sometimes she still flashes up? That you can recognize her as <oh that’s my dear mother who took care of me>, but in principle, I don’t want to say that she is nothing but a burden to me now, that I only do this as some kind of duty, that’s not the case either. Honest love is still there, but actually, due to the dementia my mother is not, um, that’s not my mother.*

*(A2, daughter, secondary carer, non-cohabiting)*

At the time of the interview, the prospective negotiation process has resulted in *acceptance* of the care situation, but this does not preclude experiencing some degree of carer stress. Nevertheless, taking everything into consideration, carers feel content in terms of the influence caring for a person with dementia has on their lives. This is a result of defining and preserving individual boundaries that prevent personal life from being restricted by informal care. Furthermore, providing care for a relative with dementia can be perceived as enrichment for their personal life which manifests itself in the form of increased family cohesion or personal growth.

*It’s of value for me to be here just because I get to spend quality time with grandmother, of which I don’t know how much longer I will have, and I can also relieve some of the burden of my grandfather, who I love above all, which also gives me a good feeling; to be able to give something back to the person who has already done a lot of good to me, […] through my grandmother, the understanding developed that, […] people in need of care need to be helped, because they cannot help the fact that they need care. And we always get a lot, and it is important that we also give and in my opinion everyone is better off for it, if you have this give and take with each other; it makes me very happy to know that I have helped, because I was also helped.*

*(D2, granddaughter, secondary carer, non-cohabiting)*

### Variants of process type 2

Although this process type incorporates three different types with contrasting orientations towards the negotiation of their own needs against care demands, they have a similar baseline situation. They all went through a crisis, which is characterised by an imbalance between providing informal care and the personal life. This crisis is a process that is not linked to a specific point in time or event. It manifests itself as a perceived restriction of one’s own needs, as well as in a perceived inability to arrange care (e.g. unfulfilled desire for greater involvement of family members), or to live their own life according to their own wishes and ideas.

*Here at home it’s like what is said would fill five lines on an A4 page and these five lines are repeated; it basically is like <When is there something to eat? I need to go to the toilet, can I lie down? Can you cover me?>* ***That*** *is what the communication is during the day and that’s a nightmare I used to have a brain that I could use.*

*(B1, daughter, primary carer, cohabiting)*

These restrictions refer to a loss of self-determination, a limitation of privacy, a reduction of work hours, a strained family climate or a high burden due to the constant mental and physical presence of care.

*This always needing to be ready, and always being alert, like have we thought of everything? Is everything organized? Will it work? […] So constantly thinking like this makes it really difficult for me at the moment. Thinking on all these levels is very stressful for me at the moment. And never forgetting anything, because then somehow it becomes a vicious cycle. […] The spontaneity is gone because of it. This made things really difficult for me. I knew on 1* ^*st*^ *May ((sigh)) as I walked home with my mother-in-law in a wheelchair from the May Festival […] and then really just cried in the last few meters because I thought <So and the other families are cycling with their children and whatever and […] I am now stuck with the mother-in-law and I can’t be spontaneous.>*

*(F1, daughter-in-law, primary carer, cohabiting)*

In contrast to process type 1, the carers in this type tend to be less advantaged regarding pre-existing knowledge of care administration or a harmonious relationship between family members which seems to additionally contribute to the perceived imbalance between caring and personal life. Carers of this type can be contrasted in terms of how they deal with the crisis, which is described as separate process subtypes in the following paragraphs.

#### Process type 2a: ‘Acting strategic is making it work’

Process type 2a comprises three primary carers (two adult-children and one spouse) who cohabitate with the person with dementia. Although the intra-family distribution of responsibilities is unshared or unequal, they are in agreement with the perceived intra-family relationship quality (Neubert et al., 2020b). The negotiation process of this type is *strategic*. The carers are oriented towards the necessity of (external) help and develop strategies which aim to achieve a balance between care responsibilities and personal needs. Similar to process type 1, they are oriented towards adapting their own demands to the care situation and taking the situation calmly with acceptance, managing the care situation (organising and monitoring care) as best as they can given the circumstances. Although the latter comes along with additional effort, in terms of coordinating formal care or assisting the person with dementia in everyday life from a distance by phone, it also provides some degree of assurance or security in terms of being able to plan one’s own time. This, in turn, enables the carer to resume personal activities while at the same time knowing that the person with dementia is well cared for.

*By 1 p.m. at the latest I check whether he has eaten something, because unfortunately that also disappears in the draw or cupboard and is forgotten. [...] And so what I do is call from work […] and check about breakfast, and lunch, call once in the morning, once in the afternoon, at 10 he [person with dementia] might like to open his balcony door and air out the place. This, I coordinate everything, also when on the go.*

*(C1, daughter, primary carer, cohabiting)*

They are able to deal with the situation calmly by approaching the situation with humour and by learning self-care and situational balancing between priorities. These strategies help to restore the balance between care and personal life which, as described earlier, does not necessarily mean that the responsibilities are shared equally over time. The crisis is thus mitigated so that the result of the negotiation is an *acceptance* of the situation as in process type 1.

*For me, of course, my freedom of movement increased extremely. And I can now work across the country again. […] It’s a huge relief for me. […] (Before I didn’t) know what I can or may do or without having permanent guilty conscience. When an ambulance drove past somewhere with flashing lights and a siren, my neck hair used to bristle and it was a burden but now with the [24-hour care assistance] my mind is clear and my neck is relaxed, and in this respect, I am now positive about the future.*

*(D1, spouse, primary carer, cohabiting)*

#### Process type 2b: ‘Reflect the situation, deal with it and pull it through’

In this type, which is made up of two adult-child primary carers, the negotiation is focussed on the emotional processing of the crisis. Factors that contribute to the imbalance between personal needs and care demands and influence the negotiation between them are a conflict-loaded relationship to other family members and the unshared or unequally shared distribution of responsibilities between family members (Neubert et al., 2020b). The carers are oriented towards the need for (psychological) help and self-reflection to, for example, understand the nature of dementia and the reasons for their overload.

*Then you can’t really; I’ll say it like this, above all if you don’t reflect and process, so that means ((breath)) such care also needs a stable person, I think; and if not then you break down with such care, […] I absolutely and totally urgently need more sessions [psychotherapy] because there is so much I want to get off my chest and want to understand why are they all leaving? Why am I standing here alone with my mother, although she is also the mother of my brothers and siblings?*

*(E1, daughter, primary carer, non-cohabiting)*

As a result of this *reflective* negotiation, they develop resilience, which manifests itself in the acceptance of the situation and the fact that the person with dementia’s life is coming to an end, in breaking off contact with people who have disappointed them and in making of pleasant plans for the future.

*When everything is over, I’ll vacate this house […] and take my assets […] and send myself to Portugal […] and that I have to say is a prospect that really makes me happy. ((laughs))*

*(B1, daughter, primary carer, cohabiting)*

Although the negotiation process is primarily internal, they develop practical strategies similar to process type 2a (e.g. use of external help which allows them to take time for themselves). At the time of the interview, the result of the negotiations manifests itself as *withstanding* the situation while knowing that this (rather strenuous) situation is soon coming to an end (e.g. due to the death or institutionalisation of the person with dementia). Thereby, being future-oriented helps them withstanding the situation. Moreover, they (also) see their caring experience as an opportunity for personal growth.

*The bad always has something good and only through difficult things I think that you grow properly ((breath)). It saves me too, that this is my attitude. […] I mean every person handles […] well I think the more you reflect, the more you can come to terms with things and the better you can deal with particular situations. That is something I have been doing intensively. Half a year ago you would have rather seen me as a spiteful person, […] because I also felt that way. Now I would say I’ve turned the corner but that was a difficult path.*

*(E1, daughter, primary carer, non-cohabiting)*

#### Process type 2c: ‘There is no alternative, you have to live with it’

In process type 2c, the carers (two adult-children, one daughter-in-law and one grandchild) are majorly cohabiting (in three of four cases), but are not necessarily the primary carer (*n*=1). They are oriented towards barriers in terms of external and internal conditions which *limit* their possibility to negotiate their own needs against the demands of care. Similar to process type 2b (‘Reflect the situation, deal with it and pull it through’), a conflict-loaded relationship with other family members and unequally or unshared distribution of responsibilities are factors, among others, contributing to their limited ability to negotiate their personal needs against the care demands (Neubert et al., 2020b). On the one hand, the barriers are evident in the lack of alternatives with regard to a satisfactory care arrangement or in the form of inner strife.

*Always being torn between being downstairs [with the PWD] but also here doing right by the children and my husband. That is something very difficult […] then also workwise because there I am @(paid)@ […] there I have to perform well as well, I can’t show up and say <That was a tough night> or <I can’t be bothered>.*

*(F1, daughter-in-law, primary carer, cohabiting)*

On the other hand, the carers either feel limited in their own ability to interact with the person with dementia or job obligations preclude the possibility of taking a greater part in the care of the person with dementia.

*So I am usually in the pharmacy for ten, eleven hours […] that means I leave at 07:30 and am back home at 19:45, 20:00 […] I then often stop by [the PWD] if my sister ordered some medication or whatever. I just always have the feeling that I just need to take a quick look here, but then I also, of course, sometimes have the desire to go back home and eat something or whatever, you know.*

*(B3, daughter, secondary carer, non-cohabiting)*

These barriers make it impossible to solve or mitigate the crisis. Instead, they *endure* the situation but know that the situation either has to change in the future (e.g. by handing the care over to formal carers) or that they still have to emotionally process recent changes in the situation (e.g. breaking off contact with siblings who refuse to participate in the care or employment of a 24-h care assistant against their own preferences).

*Of course you have say, I think more realize, that for certain things this 24-hour assistant is pretty good […] but for me it was just hard, I mean really ignorance you know, towards me, just to leave me like this, without even once thinking. You know, what is our brother doing actually, how is he going? […] That was for me, for me personally really bad in itself […] because I would never have expected that [...] I went into that conversation [with his three sisters][…] and said, ok […] we’ll find a solution. Of course we found a solution [employment of a 24-hours care assistant] but that was a solution that my three sisters wanted [...] so now we have to live with it. But the way it came about, that was very ugly.*

*(F2, son, secondary carer, cohabiting)*

Consequently, the strain is shifted rather than relieved (e.g. higher independence as a result of employing a 24-h carer but, at the same time, experiencing privacy restrictions).

## Discussion

In this study, a typology which contributes to the understanding of how family carers of people with dementia perceive the reconciliation of informal care and their personal life was reconstructed using a qualitative constructivist approach. The four process types – ‘Respect your boundaries, stay calm, but plan ahead’, ‘Acting strategic is making it work’, ‘Reflect the situation, deal with it and pull it through’ and ‘There is no alternative, you have to live with it’ – are mainly based on (1) different forms of negotiating one’s own needs against the care demands throughout the course of care (prospective, strategic, reflective and limited) which ultimately results in (2) different attitudes towards reconciling informal care and personal life (accepting, withstanding and enduring).

The process types identified do not claim to be complete, nor are they to be regarded as finished. Our findings, which are based on retrospective narratives, support those of previous studies that described caring for a person with dementia as dynamic processes that typically have no clear starting points and continuously have to be adapted (Benson et al., 2019; Bressan et al., 2020; Huis In Het Veld et al., 2016; Lopez Hartmann et al., 2016). This is related to the changes in mood and behaviour of the person with dementia which increase as the disease progresses and thus lead to the need to constantly renegotiate one’s own needs against the care demands (Huis In Het Veld et al., 2016). We therefore hypothesise, for example, that a future change in the care situation can trigger the experience of a new ‘crisis’ in carers who were able to develop strategies which enabled them to successfully adapt to the adversities until they succeeded in accepting the situation (like it is shown in the ‘Acting strategic is making it work’ type). This new ‘crisis’ may be negotiated differently depending on individual internal and external resources and thus may result in a different attitude towards reconciling informal care and personal life. However, this hypothesis has to be tested in longitudinal studies.

Many carers tend to have difficulties balancing the demands of different areas of life (Rayment et al., 2019). In the present study, some carers, mainly (but not exclusively) primary carers cohabiting with the person with dementia and with an unequally or unshared intra-family distribution of care tasks, experience a crisis of imbalance in terms of perceived restrictions in pursuing their own interests. Hence, previous findings which found that primary carers tend to have higher subjective burden are supported (Gonçalves-Pereira et al., 2020). In accordance with the findings by Varik et al. (2019), the personal life of carers experiencing a crisis is (type ‘There is no alternative, you have to live with it’) or was for some time (types ‘Acting strategic is making it work’ and ‘Reflect the situation, deal with it, and pull it through’) almost completely determined by the demands related to the care situation. Restrictions in privacy and in the fulfilment of personal needs are important factors of carer burden (Ransmayr et al., 2018). Although the life outside of care may create additional load, since other roles and responsibilities pose additional demands on a carer’s resources, it is an important resource for resilience (Teahan et al., 2018). In this sample, some primary carers (type ‘Acting strategic is making it work’) were able to mitigate the crisis and to restore an acceptable level of balance between care and personal life. Waligora et al. (2018) synthesise in their systematic review that self-care is a challenging aspect to caring, but participating in activities independent of the person with dementia is an important self-care behaviour.

As the different process types show, the carers differ regarding their success in restoring (an acceptable level of) balance between care and their personal life. Depending on internal and external factors, it is more or less challenging for them to meet their own needs and to pursue their own interests. Even though they are aware of the importance and benefits of meeting their own needs (Rayment et al., 2019), the cared-for person’s needs are often prioritised out of a sense of responsibility. Those who struggle the most and thus are the most challenged are probably carers who are limited in their abilities to negotiate their own needs against the care demands due to internal and external conditions and are consequently enduring the situation (type ‘There is no alternative, you have to live with it’). This kind of being limited can be seen as curtailment of their autonomy or control over the situation which was found to be associated with emotional stress and physical strain (Schulz et al., 2012). In contrast, process type 1 carers (‘Respect your boundaries, stay calm, but plan ahead’) have a high degree of autonomy and control by setting limits regarding the tasks they are willing to take on and the level of influence that care may have on their personal lives. This high degree of control enables them to prospectively balance their own needs against the care demands. However, since process type 1 carers are mainly secondary carers, it can be assumed that having control over the level of influence which caring has on their lives is much easier for them than for primary carers who usually spend more time with the person with dementia. Although process type 2a carers’ autonomy was curtailed in the past as well, they were able to restore their autonomy to a large extent which they achieved through the development of practical strategies as well as strategies that increase the ability to deal with the situation personally (‘Acting strategic is making it work’). This is in line with Lloyd et al. (2016) who concluded that the ability of carers to identify positive aspects of care is at least partly the result of their choices and strategies. Our findings furthermore support the hypothesis that even highly challenged carers can draw positive aspects from care (Campos et al., 2019; Joling et al., 2016; Pendergrass et al., 2019; Pristavec, 2019) as can be observed in the carers of process type 2b (‘Reflect the situation, deal with it, and pull it through’) who became resilient and recognise the challenging experience as an opportunity for personal growth (Lloyd et al., 2016). Overall, it seems that carers are capable of achieving a personal gain out of care as long as they are able to negotiate their own needs against the demands of care.

Since informal carers constitute an important pillar in the care of the older population, maintaining the willingness to provide care for relatives is a political objective (OECD, 2018). However, this will only succeed if it is possible to reconcile care with other areas of life. In the present study, carers who succeed in (re)balancing their caring role with their personal life (process types 1 and 2a) achieve this. The strategies used by these carers are consistent with those found to be beneficial in other studies. Lowering their own standards of care (not being a perfectionist), humour, understanding the importance of self-care and setting limits on the scope and activities of care were found to be efficient in maintaining or restoring balance between care and personal life (Huis In Het Veld et al., 2016; Waligora et al., 2018; Yu et al., 2018) and should thus be supported in interventions. However, there is no one-size-fits-all approach. Individual carers have different needs, resources (financial, social, network and coping skills) and live under individual conditions that affect the ability to balance care and personal life. In relation to the results of this study, this could mean that different types of negotiation benefit from different types of support which emphasises the need to identify the individual needs of carers in order to provide them with adequate support (Zwingmann et al., 2019). Since this study has shown that context factors related to the care network, such as intra-family relationship quality and the distribution of care tasks, influence the way carers of a person with dementia reconcile care with personal life, supportive interventions should not be tailored exclusively to the primary carer but should also take the perspectives and needs of other network members into account.

### Limitations and future research

Using narrative interviews, we were able to identify different patterns regarding the way carers balance their own needs against the demands of the care over time. However, the way past experiences were described is influenced by the orientation frameworks which are present at the time of the interview (Nohl, 2017). Moreover, our results are based on a relatively small sample of 14 informal carers from Germany who were recruited via gatekeepers at counselling centres. We cannot rule out that people who look for help at these kinds of institutions have specific characteristics or motivations which influence the results, nor can we exclude that gatekeepers selected specific informal carers. Furthermore, the majority of our sample are children or grandchildren of the cared-for family member who live in an urban or suburban environment. Thus, spousal carers and those living in a rural area are under-represented. However, we assume that the issue of reconciling care with personal life is especially relevant for child and grandchild carers since they are the ones who probably have a high number of responsibilities beyond care (e.g. employment or childcare). Although the documentary method involves a comparative analysis through which methodological control and generalisability is intended to be achieved, the limitations mentioned above nevertheless limit the transferability of our results. A future research direction could be to expand the typology, for example, by comparing spouses with adult-child carers regarding their way of negotiating the care demands against their own needs. An aspect which we did not focus on in this study, but has been shown to influence the perception of care in previous studies, is the relationship quality or closeness between the person with dementia and the carer (Daley et al., 2017; Gallagher & Beard, 2020; Keady & Nolan, 2003; Kaplan, 2001). As research in this area has been focussed on spousal relationships, it would be interesting to see how perceived relationship closeness between, for example, adult-children and the person with dementia factors into the processes of negotiating care demands and personal needs and the resulting attitudes towards reconciling care and personal life. Such research objective would require also taking the perspective of the person with dementia into account. Since the primary aim of the project of which this study was part was not to investigate the quality of the relationship between the MmD and the carer but to examine the reconciliation between caring for a person with dementia, family and employment, only the carers were interviewed.

## Conclusion

This study contributes to the literature on dementia care by presenting a typology on how informal carers of persons with dementia balance care needs and personal life. By identifying different negotiation processes (prospective, strategic, reflective and limited) which result in different attitudes towards the reconciliation of care and personal life (accepting, withstanding and enduring), our findings emphasise the dynamic of caring for a person with dementia. Of most practical relevance is that different process types have different needs, resources and possibilities to reconcile care with other areas of life which are shaped by the caring context (e.g. perceived relationship quality and distribution of care tasks between the members of the care network or being the primary or secondary carer). Thus, the findings emphasise the importance of taking context factors into account in order to provide effective support since they seem to significantly influence the possibility of (re)establishing a balance between one’s own needs and the demands of care. In addition, self-care, for example, by participating in activities outside care, should be promoted as it seems to be crucial for achieving a balance between care and personal life. Future studies that verify or expand the typology by comparing how carers with different relationships with the person with dementia (e.g. spouses and adult-children) reconcile care with their personal lives or by considering the relationship quality/closeness between the carer and the person with dementia, could be valuable.

## Note

1. pause; xx emphasised; **xx** loud (in relation to the usual volume of the speaker); (xx) uncertainty in transcription, difficult to understand; ((xx)) comments or remarks on non-verbal or external events; @xx@ speaking while laughing; [xx] insertion by researchers for better understanding of the quotations without context.
